# Evaluation of the potential of ultrasound-mediated drug delivery for the treatment of ovarian cancer through preclinical studies

**DOI:** 10.3389/fonc.2022.978603

**Published:** 2022-09-05

**Authors:** Yi-Chao Wang, Jing-Yan Tian, Ying-Ying Han, Yun-Fei Liu, Si-Yao Chen, Feng-Jun Guo

**Affiliations:** ^1^ Department of Obstetrics and Gynecology, The Second Hospital of Jilin University, Changchun, China; ^2^ Department of Urology, The Second Division of the First Hospital of Jilin University, Changchun, China

**Keywords:** ultrasound, microbubble, drug delivery, chemotherapeutics, RNA therapeutics, dual-mode imaging

## Abstract

Ovarian cancer (OC) has the greatest mortality rate among gynecological cancers, with a five-year survival rate of <50%. Contemporary adjuvant chemotherapy mostly fails in the case of OCs that are refractory, metastatic, recurrent, and drug-resistant. Emerging ultrasound (US)-mediated technologies show remarkable promise in overcoming these challenges. Absorption of US waves by the tissue results in the generation of heat due to its thermal effect causing increased diffusion of drugs from the carriers and triggering sonoporation by increasing the permeability of the cancer cells. Certain frequencies of US waves could also produce a cavitation effect on drug-filled microbubbles (MBs, phospholipid bilayers) thereby generating shear force and acoustic streaming that could assist drug release from the MBs, and promote the permeability of the cell membrane. A new class of nanoparticles that carry therapeutic agents and are guided by US contrast agents for precision delivery to the site of the ovarian tumor has been developed. Phase-shifting of nanoparticles by US sonication has also been engineered to enhance the drug delivery to the ovarian tumor site. These technologies have been used for targeting the ovarian cancer stem cells and protein moieties that are particularly elevated in OCs including luteinizing hormone-releasing hormone, folic acid receptor, and vascular endothelial growth factor. When compared to healthy ovarian tissue, the homeostatic parameters at the tissue microenvironment including pH, oxygen levels, and glucose metabolism differ significantly in ovarian tumors. US-based technologies have been developed to take advantage of these tumor-specific alterations for precision drug delivery. Preclinical efficacy of US-based targeting of currently used clinical chemotherapies presented in this review has the potential for rapid human translation, especially for formulations that use all substances that are deemed to be generally safe by the U.S. Food and Drug Administration.

## Introduction

Among gynecological cancers, ovarian cancer (OC) has the highest rate of mortality with a five-year survival rate of fewer than 50% (1). Because of their asymptomatic and highly differentiated character, OC is diagnosed in the majority of patients at an advanced stage, when the tumor is difficult to completely resect or has spread to other organs (2). 90% of OCs come under the category of epithelial OC (EOC) (3) of which the high-grade serous cancer (72%) is the most preponderant. The lesser varieties of EOCs include low-grade serous cancer (6%), endometrioid cancer (10%), mucinous cancer (6%), and clear cell carcinoma (6%) (4). The clinical management of EOC includes debulking surgery of tumor mass followed by chemotherapy with platinum or taxol group of drugs (5, 6). The currently available chemotherapy largely fails to treat OCs that are drug-resistant, refractory, metastatic, and recurring. Therapeutic improvements have been achieved by targeting vascular endothelial growth factor (VEGF) by bevacizuma (7) and poly adenosine diphosphate-ribose polymerase (PARP) by Olaparib to induce apoptosis of OCs bearing BReast CAncer gene1/2 (BRCA1/2) mutation (8, 9). However, significant toxicity exists for PARP inhibitors, and therefore to overcome systemic toxicity and drug resistance encountered by the available therapies of OC, the tumor-targeted drug delivery approach is emerging as a promising research area. Ultrasonography is an indispensable tool for the diagnosis of OCs which however is not suitable for screening of OCs. On the other hand, ultrasound (US) technology is being more and more investigated for OC therapy.

Ovarian tumor is among those tumors that has a large stromal compartment with a tight perivascular cell cover and a dense collagen network, giving rise to low enhanced permeability and retention (EPR) effect (10). As a result, only limited amount of drug can reach the tumour interstitium. This challenge could be circumvented by enhancing the passive uptake of drugs by tumors *via* EPR effect. One widely used method to overcome this challenge is prolonging the circulation time of drugs by surface PEGylation thereby escaping the uptake mechanism of the phagocytic cells and reducing renal clearance. With the passage of time, due to the EPR effect and the absence of lymphatic drainage, the concentration of drugs gradually increases in tumours, eventually reaching levels that are many times greater than those in plasma (11). Drug-encapsulated nanoparticles made of liposomes, polymers, dendrimers and micelles are also extensively used for tumor drug delivery using passive uptake mode. Active targeting on the other hand makes use of various targeting ligands including monoclonal antibodies, proteins, peptides, nucleic acids (e.g., miRNA, shRNA, and aptamers), and small molecules tethered to delivery systems. Active targeting enables directing carriers of therapeutic agents to cellular targets with high payload capacity. This is achieved, for instance, by facilitating receptor-mediated endocytosis, an uptake mechanism (12–15).

US could be a useful strategy for improving drug delivery to the tumors with low EPR. The biophysical effects of US on cells include cavitation, hyperthermia, and sonoporation (for an updated review (16),). Most commonly used US-mediated delivery systems include microbubbles (MBs), nanodroplets, micelles and nanoliposomes. US-responsive vehicles can undergo cavitation that could take the form of either sustainable cycle of bubble expansion and contraction or violent collapse of bubbles along with high-speed microstream (17). The thermal effects result from the absorption of acoustic energy by the tissues leading to altered cell membrane fluidity and enhanced vascular permeability (18). In sonoporation, drugs/genes enter into the cells through pores created in the cell membrane by US frequency between 0.5 to 2 mHz (19). These properties allow targeting therapeutic agents to the tumor site. In this review, we focus on the recent developments in various US-mediated drug delivery techniques aimed at improving the treatment of advanced OCs while minimizing adverse consequences.

## Principal US-mediated drug delivery systems

### Microbubbles

Given the advantages of the formation of cavitation nuclei under US sonication, MBs serve as cavitation nuclei for US-mediated gene/drug delivery (20, 21). Drug encapsulation, co-injection with drug or drug-encapsulated nanoparticles, and covalent linkage with drug-carrying nanoparticles are commonly used applications of MBs (22). To become US responsive, MBs need a gaseous core, perfluorocarbon (PFC) or gas-generating compounds. US-responsive materials are generally polymeric such as poly(lactic acid) (PLA), PFC, polyvinyl alcohol (PVA), poly(lactic-co-glycolic acid) (PLGA), and perfluoroctanol-poly(lactic acid) (PFO-PLLA) (23). The typical MBs (size, 1–10 μm) have gaseous cores and phospholipid, polymer, or protein exterior shells that move through the bloodstream (24). In the circulation, when MBs are exposed to US, they oscillate (rapidly change in size and shape/cavitation), and generate high shear stress on the surrounding endothelium causing vascular deformation, rupture and altered permeability. As a result, therapeutic substances are delivered specifically to the insonation area in a spatiotemporally regulated manner by increasing vascular permeability and endocytosis by the tumor cells. The ultimate outcome is enhanced delivery of the therapeutic substance to tumor tissue (Figure).

MBs with US-responsive materials has potential clinical application (25) and indeed several MBs have been designed for clinical trials that possess drug cargo features along with visualization attribute, and the trigger system for the release of drug at the tumor site (ClinicalTrials.gov) (https://clinicaltrials.gov/)]. However, MBs suffer from two major limitations; i) short circulatory half-life and ii) and size unfavorable for penetration to tumor tissues which only allow particles with diameter of <1μm. The tumor vasculature is rather leaky and displays obstruction in lymphatic drainage that make extravasation of MBs challenging due to their large size. To circumvent this problem, nanoparticles ranging in size between 1-100nm owing to their EPR effect efficiently penetrate the tumor tissues. Taking advantage of this feature of nanoparticles, bubbles, droplets, micelles, and liposomes with the size of nanoscale range have been designed for US-mediated drug delivery (26–28).

### Nanodroplets

Nanoscale bubbles with gaseous core (often perfluoropropane) and phospholipid outer shells have been designed with US-responsive materials to facilitate drug delivery (29). Particularly responsive to US are the nanodroplets made of volatile perfluoropentane (30). Heat or US-induced acoustic droplet vaporization can cause a phase change in it. Droplets upon stimulation by US can enlarge to become nanobubbles to enhance the US contrast and trigger the release of the therapeutic agents, generally hydrophilic drugs. Furthermore, droplets with a diameter of ≤ 200nm at 37°C are more stable than gas bubbles because of the maintenance of the liquid core in the circulation thus resisting gas dissolution. These attributes impart long circulatory time and augment the EPR effect resulting in the efficient delivery of drugs to the tumors by the droplets (31, 32).

### Micelles

Micelles are also used for US-mediated drug delivery. Micelles in most cases are self-assembling polymeric structures consisting of a hydrophilic group and hydrophobic alkanes. Given their amphiphilic structures, nano-sized micelles can encapsulate both hydrophilic and hydrophobic drugs (33). The US-induced moderate thermal effect allows extravasation of micelles to the tumor and US-induced sonication enhances the cell membrane permeability, and together these events facilitate drug delivery. US-generated shockwaves also degrade micelles and in the process promote the delivery of the drug cargo to the tumor (34). A few known block copolymers form micelles in aqueous solutions, and among them ethylene oxide-propylene oxide linear tri-block (e.g. Pluronics) gained much attention because of their ability to gene/RNA delivery under the US-mediation (35).

### Nanoliposomes

Nanoliposomes represent another strategy for US-mediated delivery given their lipid bilayer structure that aids in their better biocompatibility. However, unlike nanobubbles, nanoliposomes do not contain gas and are not very responsive to US. Nanoliposomes can be designed to encapsulate emulsions that can evaporate under US stimulation. Cavitation or heat effects of US can hasten the release of drugs or genes encapsulated in nanoliposomes (36). The thermal effect often plays the primary role in the delivery process under the high US frequency and cavitation when sonication is used at high frequency whereas cavitation is crucial when the frequency is low (37). Nanoliposomes are increasingly improved for delivering oligonucleotides and gene in response to US (38, 39). For details on the design and fabrication of a US-mediated drug delivery system based on polymeric micelles and nanocarriers, please refer to previously published reviews (34, 40, 41).

**Figure 1:**
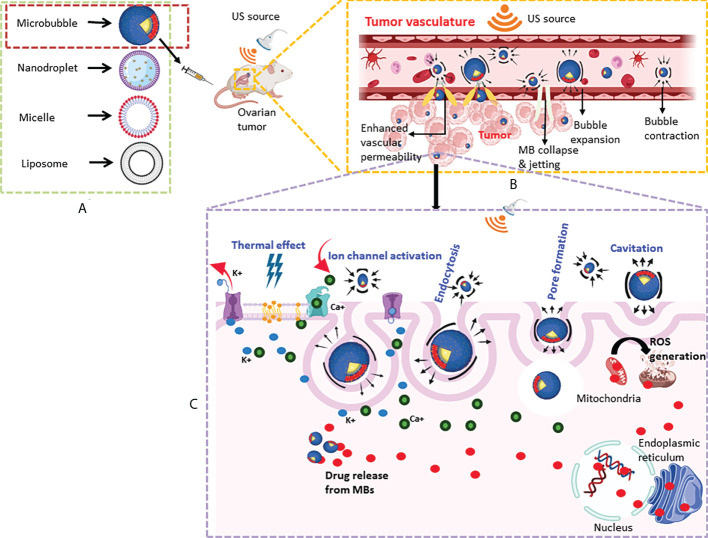
Schematic of US-mediated drug delivery system targeting ovarian tumors. **(A)** Simplified diagrammatic representation of major US-mediated drug delivery systems discussed in the review are shown (refer to table 1 for their salient features). **(B)** An example of the delivery of therapeutics to the tumor site by MBs using a US-based technique in a murine model of ovarian cancer. Circulating MBs are exposed to sonication using a focused US transducer to induce cavitation. Tumor endothelial cells become more permeable to the drug-carrying vehicles due to mechanical disruption caused by cavitation. **(C)** US-mediated changes (shown in blue) including increased membrane fluidity by thermal effect, ion channel activation, pore formation, and endocytosis promote uptake of MBs by the cancer cells. Cavitation bubbles also cause ROS generation causing killing of the ovarian tumor cells.

**Table 1** summarizes the key features of the drug delivery systems for US-mediated therapeutic targeting discussed here, which also provides an outline of the frequently used reagents on the development of these delivery systems to be discussed subsequently. Moreover, a schematic illustration of the mechanism of targeting of therapeutic substances by the various US-mediated vehicles as described above is shown in **Figure 1**. When intravenously injected, these vehicles circulate in the systemic circulation. Application of US insonation induces cavitation that disrupts the intercellular gap junction between the vascular endothelial cells of tumour vasculature. US waves also increase membrane fluidity by local tissue hyperthermia and create pores in the membrane which make tumor cells receptive for the drug-carrying vehicles. Moreover, cavitation disrupts ion channels and activates mechano-sensors to enhance endocytosis of the drug-carrying vehicles by the tumor cells. Besides, tumor cell killing is also caused by the generation of US-induced cavitation bubbles and subsequent ROS production.

**Table 1:** Salient features of the delivery systems used for US-mediated targeting of ovarian tumors and reagents used for their designing.

Delivery systems				
Name	Structural features	Common use	Advantages	Disadvantages
Microbubbles	Contains a gas core stabilized by a shell comprised of proteins, lipids or polymers with diameters ranging from 0.5 to 10μm	Delivery of small molecule drugs/biolo-gics to target tissues with or without US	Precision targeting that could enhance therapeutic index of caner chemo-therapeutic drugs and overcome drug resistance	Optimization of shell materials is tedious.
Nanodroplets	A hollow sphere of a polymer (lipidic nature) containing gas with size in the range of 1-10 nm	Transportation of pharmacologic or biological substances by escalating the rate of delivery of a specific volume of gas or fluid with or without US	High surface to volume ratio that enhances the rate of adsorption and the kinetics of reaction is accelerated; 3D structures could be fabricated; and precision targeting.	Generally unstable unless polymers such as PLGA or chitosan are used; payload is limited.
Micelles	Self-assembling polymeric structures (size, 10-100nm) with core/shell structures formed by amphiphilic block copolymers	Effective for poorly water soluble drugs	Increases blood half-life and protects against off-target toxicity of chemo-therapeutic drugs.	Poor drug-loading efficiency, and Pluronic rapidly degrades *in vivo*.
Nanoliposomes	Nanoscale bilayer lipid vesicles having small sized vesicles of 20-100nm and large sized vesicles >100nm	Oligonucleo-tide and gene delivery	Encapsulate hydrophilic and hydrophobic substances individually or at the same time	Less responsive to US; poor storage stability; and small vesicle sizes and high surface energies result in strong van der Waals forces leading to strong attraction between nanoliposomes.
Commonly used reagents for designing the delivery systems				
Name	Structural features	Common use	Advantages	Disadvantages
PLGA	Synthetic aliphatic polymer with a polyester backbone that is formed through the copolymerization of lactic and glycolic acid monomers	Control release of small molecule drugs, peptides, and proteins	Biocompatible, biodegradable and has sufficient mechanical strength	PLGA matrices are not efficient in release of proteins.
PEG	Synthetic polymer	Biocon-jugation and increasing the blood circulation time	High solubility in aqueous medium, biocompatible and good tolerance	May cause development of immunogenicity.
Liquid PFC	Synthetic fluorochemicals in which all H atoms are replaced by F.	It has high specific gravity, moderate surface tension, low viscosity, and optical clarity and transparency that make it suitable for vitroretinal surgery	Good oxygen carriers and can be vaporized using acoustic or optical droplet vaporization methods (phase changeable)	Reduces platelet counts and causes allergic reaction.
Alginate/PFH nanodroplets	Alginate (natural polysaccharide) has high gelling property and high stability. PFH for oxygen loading.	Easily gets converted into MBs under the action of US at sufficiently high rarefaction pressure	Drug release system	Achieving high pressure for MB conversion from nanodroplets may be challenging.
ICG	A photosensitive dye with considerable absorption and fluorescence in the NIR wavelength region	ICG at NIR illuminates tissues. Upon absorption of the light by the tissue, US waves are generated by thermoelastic expansion. US transducers then receive the waves to reconstruct the tissue images.	Safe and US FDA approved. It has a deeper tissue penetration as light scattering in NIR region is lower than in the visible wavelength.	Displays photobleaching tendency, poor aqueous stability *in vivo*, concentration-dependent aggregation and unstable optical properties.

PLGA, Poly lactic-co-glycolic acid; PEG, polyethylene glycol; PFC, perfluorocarbon; PFH, perfluorohexane; NDs, nano droplets; ICG, indocynanine green; NIR, near infrared.

## Passive targeting of OCs by US

Preclinical studies have indicated that passive targeting of OCs by US mediation is effective with chemotherapeutic drugs. Because gold nanocones (AuNC) have US-active qualities, cisplatin (Cis), the first line chemotherapy for ovarian tumor was mixed with AuNC and administered to a cisplatin-resistant OC line (A2780cis) in a spheroid 3D culture system with or without US. Cisplatin (Cis), the first-line chemotherapy for ovarian tumours, was combined with gold nanocones (AuNC), which exhibit US-active properties, and given to a cisplatin-resistant OC line (A2780cis) in a spheroid 3D culture system with or without US. In A2780cis, a triple combination of US + AuNCs + Cis showed anti-cancer efficacy when compared to cells without US exposure. This triple combination therapy decreased the growth of A2780cis cells by 60% in 2D culture and colony formation by 83% in 3D culture (42). In another study, doxorubicin (Dox), another chemotherapeutic drug, and curcumin, a chemosensitizer were co-encapsulated into US-responsive alginate/perfluorohexane nanodroplets (Dox-Curcumin-nanodroplets) with high entrapment efficiency. Drug release was enhanced from the designed nanodroplets by US; low frequency (28 kHz) sonication caused increased acoustic cavitation leading to higher US-induced drug release. At 28kHz, nanodroplets were visible as contrast MBs in US images that could be extremely effective for image-guided precision therapy for cancer. Moreover, mice harboring adriamycin-resistant A2780 cells when treated with Dox-Cur-ND and exposed to US displayed a high-efficiency tumor regression compared to Dox-Cur-ND without US. From these data, it appears that US-responsive smart nanodroplets such as that used here hold promise for OC treatment (43). Although, the chemosensitizing attribute of curcumin synergized the Dox effect on tumor regression, however, the combination affected the complete release of Dox from the nanodroplets. Notwithstanding this limitation, this study demonstrated the ability of US-mediated drug delivery as proof of concept for targeting OCs.

**Figure 2:**
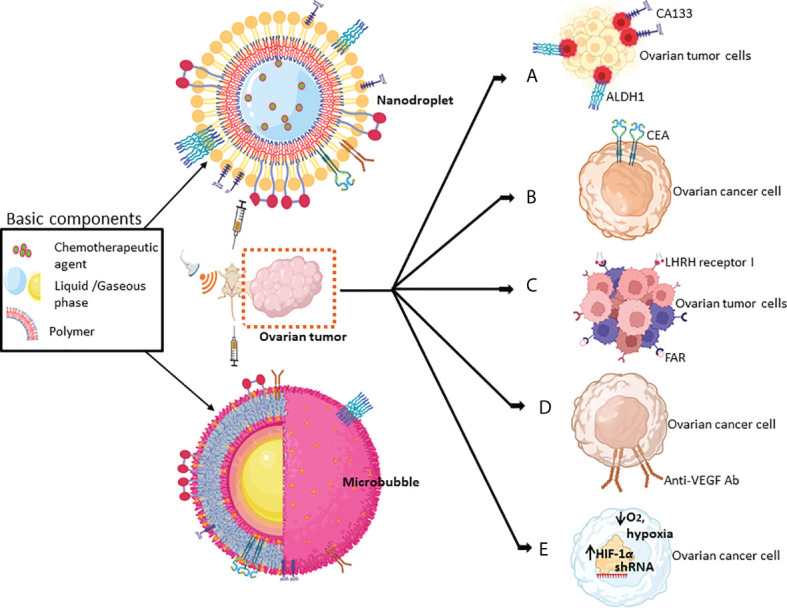
Schematic of the key strategies for targeting various biomolecules of ovarian tumors by MBs or nanodroplets mediated by US-based technologies. Chemical drugs/biologics that have been used for targeting biomolecules include **(A)** CD133+ or ALDH1+ in ovarian cancer stem cells (in red); **(B)** CEA, the surface marker of OCs; **(C)** LHRH receptor I and FAR; **(D)** the angiogenic growth factor, VEGF; and **(E)** the hypoxic responsive protein HIF-1α (refer to **Table 3** for more details).

For improved anti-cancer efficacy and safety, however, targeting protein moieties that are specifically upregulated in OCs, tumor microenvironment that becomes more hypoxic, and ovarian cancer stem cells (CSCs) offer an exciting possibility for US-mediated delivery of drugs/genes, and are discussed below. For details on OC lines most frequently used in the studies discussed here, refer to **Table 2**. A schematic diagram for helping the readers to comprehend the approaches described below (sections 4-9) for targeting the biomolecules that are elevated in the OCs and ovarian CSCs, and altered tumor tissue microenvironment is shown in **Figure 2**. Additionally, a synopsis of these strategies is presented in **Table 3**.

**Table 2:** Characteristics of ovarian cancer cell lines frequently used for various studies discussed here.

Cell line	Morphology	Histological characterization	Original source	Grade
A2780	Round	–	Tumor tissue	–
SKOV3	Epithelial	Serous	Ascites	1/2
OVCAR3	Epithelial	High-grade serous	Ascites from relapsed disease	3
OVCA433	Epithelial	Serous adenocarcinoma	Tumor tissue	–
ID8	Epithelial	Ovarian surface epithelial	C57BL/6 murine tumor tissue	–

Except ID8, all are human-derived.

**Table 3:** Strategies for targeted delivery of small molecule drugs/biologics to ovarian cancers by US mediation.

Name	Delivery system	OCs assessed	OT models used	Advantages/lacuna
CEA	PTX-loaded phase-shifting PLGA nanoparticles with PFP (PTX-PLGA-SA/PFPs)	SKOV3	–	Suitable for killing OCs at early stage. Suitability for attenuating metastatic OCs not studied.
LHRHa	FITC-labeled LHRH-a/CPT-11/PLGA microspheres PTX-loaded anti-LHRH receptor targeted phase-transformation lipid nanoparticles Lipid MBs conjugated with LHRHa *via* an avidin-biotin linkage and mixed with the pEGFO-N1-wtp53 plasmid LHRHa-targeting PTX-loaded lipid MBs *via* avidin-biotin conjugation Lipid MBs bind to LHRHa targeting survivin shRNA *via* avidin-biotin conjugation Lipid MBs bind to LHRHa targeting livin shRNA *via* avidin-biotin conjugation	A2780/DDP A2780 & OVCAR3 A2780/DDP A2780/DDP A2780/DDP OVCA-433	Balb/c xenotransplanted with A2780/DDP Balb/c xenografted with OVCAR3 - Nude mice xenografted with A2780/DDP Nude mice xenografted with A2780/DDP -	Suitable for killing OCs at the primary site as well as highly invasive types. The efficacy in metastatic condition has not been assessed.
FAR	FA surface-conjugated US-activable with PFP in the core and HMME in the phospholipid shell FA surface-conjugated nonocage with curcumin, ferritin and PFH in the core (stimulation by LIFU) HCPT containing NDs with FA in the surface and Fe_3_O_4_ and PFP in the core (stimulation by LIFU) FA-lipid-PLGA nanoparticle containing SIK2 siRNA and antisense miR21 in a layer-by- layer structure to protect RNAs from degradation miR-34a-mimic-FA-MBs (stimulation SKOV3 by UTMD)	SKOV3 SKOV3 SKOV3 OVCAR3, A2780, and SKOV3	Nude mice xenografted with SKOV3 Nude mice xenografted with SKOV3 Nude mice xenografted with SKOV3 Nude mice xenografted with OVCAR3	Suitable for killing OCs at the primary site. Suitability for attenuating metastatic OCs not studied.
CSC	Apoptosis induction and stemness reduction by LIUS GSH-responsive PSP@MB for sonoporation and endocytosis of ALDH1 shRNA in CSCs	CSCs from OVCAR5 and A2780 CSCs (ALDH1+ & CD133+) from fresh human ovarian tumor cells	Cis+LIUS reduced CD133+ cells in OVCAR5 and A2780 cell-harboring xenograft mice Primary human ovarian tumor cells xenografted into female NOD.SCID mice	Killed specific subpopulation of ovarian CSCs and suppressed ovarian tumor growth. Efficacy in preventing relapse not studied.
Angiogenesis	VEGF siRNA-complexed ABP was then combined with US for activating MBs	A2780	Nude mice xenografted with A2780	Effective in killing OCs at the primary site. Anti-metastatic potential not assessed.
Hypoxia	PFC (artificial oxygen carrier) loaded in lipid MBs for US-mediated therapy [(PTX/ICG) and oxygen-loaded PLGA nanoparticles PIO_NPs]; PFP core for carrying oxygen and a PLGA shell ICG and PTX	PTX-resistant SKOV3 SKOV3	Nude mice xenografted with SKOV3 Nude mice xenografted with SKOV3	Effective in killing drug-resistant OCs and likely suitable for killing CSCs.
Immunogenic cell death	NPs loaded with oxaliplatin ICG (a photo-/sonosensitizer), and PFP (that undergoes phase transition under US treatment)		Syngenic mice xenografted with ID8 tumor cells	Induction of strong cytotoxic T cell response to murine tumor cells. ICD in human OCs has not been assessed.

CEA, Carcinoembryonic antigen; LHRHa, leutinizing hormone releasing-hormone agonist; FAR, folic acid receptor; CSC, cancer stem cell; ALDH1, aldehyde dehydrogenase 1; ABP, arginine-grafted bioreducible polymer; PTX, paclitaxel; Cis, cisplatin; PFP, perfluoro-n-pentane; PIO_NPs, PTX/ICG and oxygen loaded PLGA nanoparticles; LIUS, low intensity ultrasound.

## US-mediated drug delivery using tumor markers

### OC-specific biomarkers

Biomarkers for the diagnosis of OCs include CA125 and human epididymis protein 4 (HE4). Individually, none of these predict early-stage OCs, however, based on Risk of Malignancy Index and Risk of Ovarian Malignancy Algorithm, simultaneous measurement of CA125 and HE4 levels could be more accurate in predicting the risk of OCs in patients with suspected benign ovarian tumors (44). Carcinoembryonic antigen (CEA), which increases in both colon cancer and OCs (45, 46) is commonly used in gynecological cancer screening. Zhou et al. fabricated molecular US imaging and chemotherapy by targeting CEA (47). To this aim, a two-step targeting strategy was employed; i) in the first step, PTX-loaded heat-induced phase-shifting PLGA nanoparticles with perfluoro-n-pentane (PFP) were prepared and ii) in the second step, PTX-PLGA-streptavidin/PFPs and PTX-PLGA-anti-CEA antibody/PFPs were prepared. The tumor target/non-target (T/NT) ratio can be effectively improved using pre-targeting technologies. Pre-targeting techniques have a substantially greater T/NT value than direct targeting strategies (48, 49). However, US-mediated molecular targeting has been seldom used as a pre-targeting technique. The rationale for using PLGA was its ability to pass through the endothelial space of tumor neovascular bed and that for PFP was low-intensity focused US-mediated (LIFU) ablation of SKOV3 OCs. In this two-step process, the anti-CEA antibody targeted CEA on the surface of the human OCs (SKOV3 cells), and then PTX-PLGA-streptavidin/PFP nanoparticles were covalently bound to the anti-CEA antibody. Upon irradiation at 7.5 W, the PTX-PLGA-streptavidin/PFPs complex enhanced the cancer cell visibility by US imaging. Moreover, with the rise in temperature due to irradiation as well as LIFU resulted in the phase-shifting of PTX-PLGA nanoparticles into MBs, thereby augmenting the efficiency of the killing of SKOV3 cells (47). This technique thus offers a dual function strategy of US-based imaging and cancer cell killing.

### Luteinizing hormone-releasing hormone receptor

Although not considered a specific marker of OC, the LHRH receptor is expressed in various cancer cells including OCs but not in healthy cells allowing for precision targeting of OCs (50). Several US-mediated techniques for precision delivery of chemotherapeutic drugs and biologics (shRNAs) in nano-carriers and MBs have been developed to increase their efficacy and reduce adverse effects as exemplified subsequently. Camptosar/irinotecan (a selective topoisomerase-1 inhibitor), used as the second-line chemotherapy drug after the development of resistance of platinum-based drugs in OCs showed serious adverse reactions in many patients (51). To reduce the severity of side effects of the drug, Zhu et al. fabricated novel Camptosar-loaded nanoparticles with PLGA material that was connected with LHRH peptide or LHRH agonist (LHRH-a/camptosar/PLGA) (52). In a highly invasive human OC line (A2780/DDP), LHRH-a/camptosar/PLGA by US mediation more effectively inhibited growth and induced apoptosis compared to LHRH-a/camptosar/PLGA without US mediation. Consistent with *in vitro* results, in nude mice bearing subcutaneous A2780/DDP tumors, the LHRH-a/camptosar/PLGA bearing nanoparticles under US sonication strongly inhibited tumor growth by inducing apoptosis, and suppression of tumor angiogenesis. In another study, taking advantage of high LHRH receptor expression in OCs, biotinylated LHRH peptide (LHRHa) and avidinylated MBs were mixed with wild-type p53 plasmid to achieve peptide conjugated MBs *via* the strong avidin-biotin linkage. A2780/DDP cells were then treated with LHRHa and p53-loaded MBs (fabricated MBs) by UTMD. SKOV3 cells that do not express the LHRH receptor were used as a negative control. UTMD-mediated targeting by fabricated MBs resulted in higher transfection of A2780/DDP cells than the non-targeted lipid microbubbles leading to induction of apoptosis due to cell cycle arrest (53). In another strategy, anti-LHRH receptor antibody and PTX were loaded to phase-transformation lipid nanoparticles (PTNP) to fabricate PTX-anti-LHRHR-PTNPs. When combined with low-intensity focused ultrasound (LIFU), PTX-anti-LHRHR-PTNPs led to an increase in the circulatory half-life of PTX and increased its penetration to the tumor site resulting in an efficient reduction in the size of xenograft of OVCAR-3 tumor in nude mice (54).

The studies discussed above assessed the targeting efficacy of LHRH by US-mediation in the non-metastatic OC model. Since OCs are reported to metastasize along the peritoneal lining causing metastatic lesions (55), an attempt was made to synthesize LHRH receptor-targeted and PTX-loaded lipid MBs (PLMB) for US-mediated therapy. PLMBs displayed features of the clinically used US contrast agents, and US-guided targeting selectively induced apoptosis of A2780/DPP cells (56). When PLMBs were treated to nude mice bearing A2780/DPP cells (xenografted at the i.p. site), a significant increase in survival time was observed compared to placebo-treated control mice. The anti-tumorigenic effect of PLMBs was accompanied by increased apoptosis of cancer cells and downregulation of angiogenesis. Mice treated with PLMBs did not exhibit any adverse effects. These findings imply that US-mediated PLMBs are not only capable of targeted killing of highly invasive ovarian cancer cells but also inhibit metastasis (57).

Besides precision delivery of chemotherapeutic drugs to ovarian tumors, LHRHa decorated MBs through UTMD mediation have also been used for the successful silencing of genes that favor OC growth. In this regard, survivin, a member of the inhibitors of apoptosis protein (IAP) family that is generally undetectable in normal adult tissue but shows high expression in several malignancies including OCs was silenced by shRNA. The relevance of survivin as an anti-cancer target for OCs has been shown through a meta-analysis in which survivin expression was positively correlated with the International Federation of Gynecology and Obstetrics classifications for staging OCs and could potentially become a novel clinicopathological marker of this cancer type (58). Zhang et al. used LHRHa decorated MBs to target shRNA of survivin to LHRH receptor expressing A2780/DDP OCs through UTMD mediation, and observed strong suppression of cancer cell growth with concomitant induction of apoptosis (59). Livin is another member of the IAP family that is highly expressed in developing and cancer tissues, and in OCs it promotes cancer progression by activating the transcription factor YAP (60). Xu et al. took the strategy of downregulating livin by shRNA. To this aim, lipid MBs were conjugated with LHRHa by avidin-biotin linkage and MBs were then mixed with recombinant livin shRNA plasmid. UTMD-mediated delivery of livin shRNA containing MBs targeted LHRH receptor-positive OVCA-433 OCs remarkably downregulated caspase 3 and caspase 8 thereby inducing apoptosis of these cells (61). These studies highlight the potential of selective induction of apoptosis of OCs by US-mediated targeting of LHRH receptor.

LHRH receptor has two isoforms, LHRH receptor I and –II (62). So far, LHRH- receptor I has been targeted through US-mediated strategies as discussed above, and LHRH-II and its receptor have received less attention if any. LHRH-II receptor and its splice variants are known to be expressed in OCs, and LHRH-II receptor exerts an antiproliferative effect (63, 64). Therefore, targeting LHRH-II receptor alone by US-mediated technique could offer a simple and effective strategy for the targeted killing of ovarian tumors.

### Folic acid receptor

Folic acid (FA) acts through a glycosylphosphatidylinositol-anchored cell surface FA receptor (FAR) (65) and its expression is far greater in the majority of cancers including OCs than in normal tissues (66). OC targeting by FAR show theranostic potential as demonstrated by Yang et al. (30) in a study where an FA-conjugated surface contained dual US-activable nanodroplets along with PFP in the core and hematoporphyrin monomethyl ether (HMME)-loaded in the phospholipid shell. The presence of PFP caused acoustic droplet vaporization (ADV) of the formulation when exposed to US irradiation thus causing phase transition from liquid to gas resulting in the rapid accumulation of nanodroplets. This phase transition from liquid to gas led the rapid accumulation of nanodroplets at the tumor site of mice xenotransplanted with SKOV3 cancer cells. Because of deep tumor penetration of nanodroplets and high ROS production by US irradiation at the tumor site significant reduction in tumor size in mice was observed by this FA-conjugated sonodynamic approach in comparison to control mice. Given the efficient tumor guidance, this formulation was found to be safe (30).

In another strategy, Guo et al. used the pH-sensitive ferritin as a nano-cage carrier co-loaded with anti-cancer natural compound curcumin and acoustic responsive compound fluorocarbon perfluorohexane (PFH) inside, and it was conjugated outside with tumor-targeting molecule FA to fabricate a multifunctional nanoplatform (FA-FCP). The advantages of FA-FCP include improved water solubility of PFH and curcumin, high physiological stability in different media, and showed favorable biocompatibility and biosafety *in vivo* and *in vitro*. When exposed to LIFU, FA-FCP released a large amount of curcumin and PFH at pH = 5.0 thereby allowing contrast-enhanced US imaging. Cultured human OC-derived SKOV3 cells underwent apoptosis, and tumor weight was regressed by FA-FCP+LIFU with excellent biocompatibility assessed from histopathologic assessments of vital organs (67).

Besides investigational anti-cancer drugs, clinically used chemotherapeutic drugs have also been targeted to OC. Topoisomerase I inhibitors, the second line OC therapy has severe adverse effects and for mitigating which LHRH-a targeted delivery strategy was employed (52). In this regard, FAR targeted delivery appears to hold promise in the effective and safe delivery of 10-hydroxycamptothecin (HCPT), another topoisomerase I inhibitor. To this aim, Liu et al. (68) fabricated phase-changeable and folate-containing PFC nanodroplets laden with superparamagnetic Fe_3_O_4_ and HCPT for multimodal tumor imaging and targeted therapy. PFC nanoparticles as precursors of MBs are being actively studied for US-mediated imaging and drug/gene delivery, which present many advantages compared to MBs and have become promising diagnostic and therapeutic nanoagents. Moreover, they simultaneously exhibit great potential for application in multimodal imaging. In this case, the presence of Fe_3_O_4_ in the nanoformulation enhanced magnetic resonance imaging (MRI) and photoacoustic (PA) imaging which enabled accurate guidance and monitoring of HCPT. Besides, the use of LIFU-induced sonication improved US imaging by phase-shifting (a liquid to gas phase changing capacity conferred by PFP), thereby releasing HCPT at the tumor site of mice bearing SKOV3-derived tumors and demonstrating the theranostic ability of this novel drug delivery method for the treatment of OC.

Targeting OCs by FA has also been used by Liu et al. (69) to sensitize these cancer cells to PTX *via* the silencing of salt-inducible kinase 2 (SIK2) and miR-21. SIK2 expression has been reported to be higher in cancers than in normal tissues surrounding cancer, and reduction in SIK2 expression in OCs showed anti-cancer characteristics including decreased G1/S transition, delayed mitotic progression, and decreased cell survival mechanism by inhibiting Akt activation (70). On the other hand, serum miR21 levels in patients with ovarian tumors are higher than the control suggesting its potential for diagnosis of OC (71). Knockdown of miR21 in epithelial OC (EOC) significantly increased the expression of the tumor suppressor gene, Phosphatase and tensin homolog (PTEN) with a concomitant decrease in proliferation and invasion of cancer cells (72). Given the pro-tumorigenic function of these two molecular targets, Liu et al. synthesized a folate−lipid−PLGA nanoparticle-containing two RNAs (SIK2 siRNA and antisense miR21) (designated as FaLPHNPs) and fabricated a layer-by-layer structure to protect RNAs from degradation in the circulation. FaLPHNPs displayed a sustained release of RNAs with a long-circulation profile having a t_1/2_ of ~8.5 h. US stimulation and MB-mediated sonoporation of FaLPHNPs showed significant transfection of EOC cell lines (OVCAR3, SKOV3, and A2780) *in vitro* and accumulation in EOC xenografts *in vivo*. Besides, FaLPHNPs synergized the effect of PTX in mice bearing xenograft of OVCAR3 cells compared to PTX or FaLPHNP monotherapy, suggesting a chemosensitizing effect of this technique (69). FA-mediated delivery of miR34a to the tumor site by the UTMD method also demonstrated therapeutic efficacy in OC. miR34a is frequently inactivated by CpG methylation in OC, and there is an inverse association between its expression and tumor grading thus suggesting the tumor suppressing effect of this miRNA (73). Overexpression of miR34a downregulated Notch1 and Bcl2 and upregulated caspase 3 in SKOV3 cells. miR-34a-mimic-FA-MBs when injected into mice harboring ovarian cancer and delivered to the tumor site by UTMD resulted in decreased tumor growth and increased probability of survival (74). Whereas survivin and livin genes were silenced by LHRH-a, FA was used to silence SIK2 and Notch1 genes for selective induction of apoptosis of OCs, thus suggesting promising use of US-mediated RNA therapeutics for ovarian malignancies.

FAR is not only high in OCs but also in tumor-associated macrophages (TAMs). TAMs constitute the most abundant infiltrating immune cells of ovarian tumors and ascites, and have key roles in tumor growth and metastasis (75, 76). Folate targeted and oxygen/PTX-loaded lipid microbubbles (TOPLMBs) and oxygen/PTX-loaded non-targeted microbubbles (OPLMBs) were synthesized and tested for UTMD-mediated combination therapy in a FAR positive SKOV3 cells xenografted to Balb/c nude mice. It was observed that TOPLMBs targeted both ovarian tumor cells and TAM more efficiently than OPLMBs thereby significantly enhancing the chemotherapeutic efficacy of PTX. This approach highlights the potential of dual targeting of ovarian tumors and TAM in clearing from the intraperitoneal site, which constitutes a common site for the metastasis of OCs (72).

## US-mediated targeting of ovarian cancer stem cells

Expansion of various subsets of cancer stem cell (CSC) is key to cancer relapse, and in this regard, CD133^+^ OCs display greater drug resistance, tumor metastasis, and sphere formation over CD133^−^ OCs (77). Ovarian tumors frequently metastasize to the peritoneum, where the tumor cell spheroids continue to grow and persist in the absence of substrate adherence. One of the most important characteristics of CSC is its capacity to withstand anoikis, the apoptotic program caused by the loss of anchorage property which enables the cells to metastasize. The clinical management of patients would be positively impacted by the successful eradication of CSCs (78). The clinical importance of CSC has been demonstrated by the shorter time to cancer progression and higher overall survival rates in patients with chemotherapy-sensitive CSCs than without them (79).

Gong et al. (80) observed that LIUS suppressed the survival, self-renewal, and anti-apoptosis traits of CD133 cells and the stemness genes including Nanog, Sox2, and Oct4. Further, LIUS significantly inhibited a stemness-promoting signaling pathway triggered by STAT-3 phosphorylation and its ability to bind with DNA to upregulate the expression of downstream effectors including Cyclin D1, Survivin, and S1pr1, which define the underlying mechanism of LIUS-induced suppression of survival and stemness of ovarian CSC (80). In OVCAR5 and A2780 cell-harboring xenograft mice, LIUS in combination with cisplatin strongly inhibited the tumor growth relative to cisplatin treatment alone by efficiently eliminating CD133+ CSC in the tumor. Moreover, the tumors in the mice treated with cisplatin alone relapsed quickly and grew after the cessation of treatment. In contrast, the OC-xenografted mice receiving a combination of LIUS and cisplatin displayed no tumor growth recurrence after the stoppage of treatment that attested to clearance of CD133+ CSC by LIUS (80).

Besides CD133, ovarian CSC strongly expresses aldehyde dehydrogenase 1 (ALDH1) (81) and is considered a marker of OC which makes it a chemotherapeutic target (82, 83). To target ovarian CSCs, Liufu et al (84) designed a bioreducible UTMD-triggered nanocarrier to deliver ALDH1 shRNA. For achieving the reducible goal, glutathione (GSH)-sensitive vector was complexed with polyethylene glycol-disulfide (S-S) bond-polyethyleneimine loaded microbubble (PSP@MB) that contained PEGylated polyethyleneimine nanoparticles bound by S-S bonds and lipid MB bound by a biotin-avidin bridge. Modification of PEI with PEG by S-S bonds shielded its charge and diminished toxicity and non-specific interactions with serum proteins. Although PSP has efficient gene transfection ability *in vitro*, it, however, is not so *in vivo* which is made possible by UTMD-triggerable MBs for high-efficiency delivery of ALDH1 shRNA to CSCs residing in high reducing microenvironment of ovarian tumors. US-mediated delivery of GSH-responsive PSP@MB resulted in enhanced sonoporation and endocytosis of ALDH1 shRNA in CSCs derived from A2780 cells thereby leading to apoptosis of these cells compared to appropriate control CSCs (84).

Targeted overexpression of miRNAs in ovarian CSCs by UTMD can also be a strategy for OC as it showed promise in preclinical studies. A tumor suppressor miRNA, Let-7 when overexpressed in glioblastoma cells led to their reduced growth as well as a reduction in CSC markers including nestin and CD133 (85) thus demonstrating the potential of arresting the growth of other CSCs including ovarian CSCs. Let-7b belongs to the Let7 family and has low expression in serous OCs (86). Based on these reports, Yang et al. targeted CD133+ CSCs through UTMD-mediated delivery of miR-Let-7b and observed a significant loss of growth and induction of apoptosis of these cells compared with the empty vector-transfected cells (87).

Although, the killing of CSCs provides an efficient mode of treatment, and has been addressed by the strategies described above, however, relapse of ovarian tumors is critically contributed by a subpopulation of quiescent CSCs that can be identified by the retention of the lipophilic dye PKH which intercalate in the cell membrane (88). Since no studies addressed the killing of quiescent CSCs in the ovarian tumor, premature to assert that CSC targeting as explored here can completely prevent tumor relapse.

## US-mediated targeting of angiogenesis and vascular markers

Among the pro-tumorigenic growth factors, VEGF is a major promoter of several malignancies including OCs. VEGF is overexpressed in OCs and enhances tumor tissue angiogenesis leading to tumor growth (89). Hence, suppressing the expression of VEGF by siRNA technology has a high translational potential for the treatment of OC. However, naked siRNAs are degraded by the ribonucleases present in serum and thus fail to reach the tumor site at the high levels required for its optimum activity (90, 91). Additional challenges include electrostatic repulsion between the negatively charged backbones of siRNA and negatively charged cellular membrane. One extensively used method to overcome these challenges is to deliver siRNAs by cationic polymers (CPs) such as 25 k-branched-polyethylene (bPEI) through electrostatic interaction – CPs contain positively charged amines backbone and siRNAs contain negatively charged phosphate backbone (92). As a result, polycomplexed nanoparticles, called polyplexes are formed when CPs are mixed with siRNA, and these polyplexes are protected from serum ribonuclease degradation. A major limitation of bPEI is the significant cytotoxicity posed to non-malignant cells (93). To circumvent this limitation of bPEI, Florinas, et al. fabricated arginine-grafted bioreducible polymer (ABP) for siRNA delivery. ABP polymers are biodegraded due to the reduction by intracellular glutathione disulfide bonds in its backbone resulting in the release of siRNA into the cytosol. VEGF siRNA-complexed ABP was then combined with US activating MBs that acted as cavitation nuclei to form transient membrane pores. Application of US, therefore, caused sonoporation and uptake of polyplexes. siRNA was stable for 60 min in a serum-containing medium and US-mediated transfection resulted in an efficient reduction of VEGF secretion by the A2780 line without affecting cell viability. Moreover, under the US-directed targeting, the VEGF siRNA-ABP-MB complex significantly diminished the A2780 xenograft tumor volume in nude mice. Therefore, this strategy represents an efficient non-viral siRNA delivery method to treat OC without causing off-target effects (94).

The targeting strategies applied so far to inhibit angiogenesis remained limited to VEGF. Several molecules, particularly the OC-associated vascular endothelial cell-associated transmembrane glycoproteins including endoglin (CD105) and integrin α_v_β_3_ are expressed predominantly on tumor endothelial cells that are undergoing active angiogenesis (95, 96). These glycoproteins activate signaling pathways that control gene expression, modulate cytoskeletal architecture, alter cell adhesion, and promote cell survival, consequently facilitating the ability of the OCs to metastasize and better survive in varied tissue microenvironments. Future research is needed to compare the metastatic outcomes achieved with VEGF targeting to the effectiveness of targeting endoglin and integrin α_v_β_3_ by the US-mediated methods discussed above.

Abnormal blood flow that is typical of ovarian tumors is associated with altered vascularization and is utilized for early detection of malignancy. Transvaginal color Doppler (TCD) is used that relies on the detection of abnormal blood flow indicative of the malignancy for suspected patients. However, the sensitivity and specificity of this detection method are not very efficient thus leaving the scope for improvement (97–99). Molecularly targeted contrast-enhanced ultrasound (CEUS) could potentially overcome the sensitivity and specificity issues associated with TCD thereby enabling early detection of OC. Targeting OC-associated vascular endothelial markers by CEUS is a rational strategy for efficient non-invasive imaging of OCs. In this regard, CD276 appears to be a suitable vascular marker in OC and other cancer cells (100–102). The diagnostic potential of CD276 in the ovarian tumor was assessed for which mouse vascular endothelial cells (MS1) were first stably transfected with the human CD276 gene to produce MS1_CD276_ cells and then coinjected to mice with human OC 2008 cells (endometrioid adenocarcinoma) to induce xenograft tumors (the experimental group). Mice that received 2008 cells served as the control group. For CEUS, anti-CD276 antibody-functionalized contrast MBs were used for imaging of both groups. Compared with the control group, US imaging signal was significantly higher in the mice that received MS1_CD276_ cells and 2008 cells. This study suggests that the difference in expression of CD276 on vascular endothelial cells could be exploited for US imaging of OCs (103). Whether or not the expression of other OC-associated vascular endothelial markers such as endoglin and integrin α_v_β_3_ provide improved US-based visualization should be investigated in the future. Moreover, an improved early detection as well chemotherapy delivery by US targeting of an OC-associated vascular endothelial marker could become a big breakthrough towards the early cure of ovarian tumor.

## US-mediated mitigation of hypoxic microenvironment of ovarian tumor

Altered tumor vasculature has been harnessed for the delivery of chemotherapeutic drugs to ovarian tumors. In the case of an ovarian tumor, abnormal vasculature is characterized by leaky and disorganized vessel networks resulting in poor uptake of anti-cancer drugs (104, 105). Moreover, poor perfusion of tumor tissue causes hypoxia and the hypoxia-induced acidity impairs cellular uptake of anti-cancer drugs and hypoxia increases drug resistance of cancer cells through genetic instability (106). Tumor hypoxia also reduces the anti-cancer potential of those chemotherapeutic drugs that rely on oxygen production to cause ROS-mediated killing of tumor cells (107). To overcome these issues, oxygen, and PTX-loaded lipid MBs (OPLMBs) were synthesized for US-mediated combination therapy to treat OCs wherein MBs could potentially deliver oxygen and anti-cancer chemotherapeutic drugs simultaneously. Encapsulation of perfluorocarbon (PFC) served as an artificial oxygen carrier as it has superior oxygen solubility. The destruction of MB by the US resulted in enhanced delivery of PTX and oxygen that downregulated the expression of HIF-1α and MDR1/P-gp expression in PTX-resistant SKOV3 cells consequently making the cells PTX sensitive and in turn causing growth inhibition and apoptosis induction (108). In a mouse tumor xenograft model bearing SKOV3 cells, OPLMBs followed by US mediation resulted in enhanced oxygen and anti-cancer drug delivery to tumors with consequent decreases in tumor tissue, angiogenesis, and HIF-1α and P-gp expression (109). Available reports suggest that hypoxia has a substantial impact on CSC maintenance and development (110). In this context, combined targeting of the HIF-1 and HIF-2 pathways may enhance the therapeutic responses of the ovarian tumor within its hypoxic microenvironment by depriving the CSCs of these growth-promoting factors. Future research should be directed at targeting OPLMBs to ovarian CSCs to mitigate tumor relapse.

Disorganized vasculature-induced hypoxia in OCs that causes chemotherapeutic drug resistance is overcome by photo-sonodynamic (PSDT). Indocyanine green (ICG) is a sensitizer of PSDT that responds to near-infrared (NIR) and US and has been approved by the FDA for clinical use. PFP is known to be a good oxygen carrier and also enhances US and photoacoustic signals after vaporization. Under this backdrop, Chen et al. (111) synthesized nanoparticles [(PTX/ICG) and oxygen-loaded PLGA nanoparticles (PIO_NPs] with a PFP core carrying oxygen and a PLGA shell containing ICG and PTX to improve PTX’s therapeutic index. The PIO_NPs had good optical stability and transit phase capability. Furthermore, PTX could rapidly be released and ROS generated under NIR laser or LISU mediation. The PIO_NPs enhanced contrast of the US and PA imaging. PIO_NPs under LISU increased the survival time of nude mice with SKOV3 cell-derived ovarian tumor by 78% compared with PTX alone group (50 d vs. 28 d), decreased tumor volume, and induced apoptosis of the tumor cells. By using PA imaging to observe the presence of PIO_NPs near a tumor site, PIO_NPs can be utilized for cancer imaging and therapy.

## US-mediated enhancement of immunogenic cell death effect of chemotherapeutic drugs

Immunotherapy has a transformative impact on solid malignant tumors including melanoma and renal cell carcinoma. However, ovarian tumors are much less responsive to immunotherapies (112). Notwithstanding the attenuated response of OCs to immunotherapy, a few anti-neoplastic chemotherapeutic drugs including doxorubicin, oxaliplatin, and cyclophosphamide reported inducing immunogenic cell death (ICD) (113–115). ICD is defined by the chronic exposure of damage-associated molecular patterns (DAMPs) to the immune system, which then triggers autoimmunity and promotes immune‐mediated clearance of malignant cells in the tumor microenvironment. The induction of antitumor immunity leading to ICD involves endoplasmic reticulum (ER) stress response, generation of reactive oxygen species, and secretion of DAMPs (116). During ER stress, calreticulin is produced and exported as a DAMP molecule to the cell surface of apoptotic tumor cells which are then engulfed by the macrophages giving rise to immune response through antigen presentation by dendritic cells (DCs) (117). Hence, ICD induction of anti-neoplastic chemotherapeutic drugs could potentially elicit an anti-tumor immune response akin to that caused by vaccines. As anti-neoplastic chemotherapeutic drugs suffer from poor bioavailability and display high drug-related toxicity and drug resistance that preclude escalating their doses, a photoacoustic and US imaging strategy was devised to circumvent these limitations. In this regard, nanoparticles loaded with oxaliplatin (an ICD inducer), ICG (a photo/sonosensitizer) having much greater uptake by tumor cells than non-malignant cells), and PFP (that undergoes phase transition under US treatment) were fabricated and designated as OI_NPs. When OI_NPs were laser irradiated, an instantaneous rise in temperature was caused by ICG following the absorption of photons which in turn caused PFP to vaporize thereby allowing US imaging and generation of photoacoustic (PA) signals. The anti-cancer efficacy of OI_NPs was tested on ID8 murine surface epithelial cells that induced the formation of peritoneal tumors and ascetic fluid. ID8 cells are also used as a syngeneic mouse model for studying OCs (118). OI_NPs were efficiently taken up by ID8 cells by photo-/sonodynamic therapy achieved by NIR and US resulting in the induction of cytotoxicity. Moreover, ID8-induced tumor immunity was significantly higher in mice that received OI_NPs + PSDT assessed by the delay in the tumor appearance, and also induced a stronger cytotoxic T lymphocyte activity compared with mice that received free oxaliplatin (119). A further improvement was done on OI-NPs by the same research group by fabricating a phase-adjustable core-shell nanoparticle (OIX_NPs) carrying oxygen in the core along with ICG and oxaliplatin. As deep-seated tumors have low oxygen concentration in the hypoxic core, the presence of oxygen enables the visibility of these tumors by photodynamic therapy. OIX_NP was found to be able to enhance the US/PA imaging thereby serving as a contrast agent. Furthermore, in syngenic mice bearing ID8 cells, OIX_NPs suppressed tumor growth by enhancing tumor immunogenicity, thus serving a theranostic role (120).

In some rare cases, the beneficial immunologic response could be evoked by radiation by a so-called abscopal effect in which metastatic cancer located distantly from the primary site is regressed following irradiation of the primary tumor. The abscopal effect is an immunologic response to radiation in which radiation-induced cell damage leads to increased cytokine/chemokine synthesis, which in turn activates immune cells such as dendritic cells and CD-4+/C8+- T cells (121, 122). The abscopal effect has also been reported in a Hodgkin’s disease patient treated with a monoclonal antibody (pembrolizumab) against a checkpoint inhibitory protein, programmed cell death ligand 1 (PD-1) interspersed with radiotherapy. The patient displayed a complete regression of the malignant lymph node two months after this therapeutic regimen (123). Hence, a combination of radiation with immune checkpoint inhibitors to stimulate the abscopal effect and enhance the immune response of the tumor is emerging cancer therapy. Future research can examine whether an abscopal effect induced by radiation therapy could further improve the therapeutic outcome of US-mediated targeting of OCs with anti-neoplastic drugs.

## US-mediated delivery of a tumor suppressor protein

A recent study found that some members of the sirtuin (Sit) family (Sirt1-7), which are mammalian homologs of the yeast silent information regulator (Sir2) gene, play important roles in cancer development (124). Sirts are nicotinamide adenine dinucleotide (NAD+)-dependent deacetylases that target a variety of protein substrates that has effects on oxidative stress regulation, metabolism, cell survival, division, and aging (124, 125). Among the sirtuins, Sirt3, a genomically expressed, mitochondrial-located tumor suppressor protein has an aberrantly low level of expression in various cancers including OCs (126–128) where Sirt3 has anti-proliferative and anti-migratory effects. Sirt3 gene delivery by UTMD *in vitro* inhibited proliferation, epithelial-to-mesenchymal transition, and migration, and induced apoptosis and cell cycle arrest of SKOV3 cells. Furthermore, UTMD delivery of Sirt3 in mice bearing SKOV3 xenograft tumor suppressed tumor volume and weight with an attendant decrease in Ki67 positive cells (126).

## Evaluating the translational potentials

The extensive US-mediated preclinical strategies for the improved treatment of OCs described in the foregoing sections have varying degrees of translational potential. Multimodal design strategies in which attaching multiple functional moieties such as chemical drugs/biologics and targeting agents onto the particle surface have been achieved with fewer synthetic hurdles, and resulted in significant anti-cancer efficacy in preclinical ovarian tumor models. Even though the majority of these strategies are still too early for clinical application, a few could translate quickly into the clinic, which is discussed subsequently.

Firstly, the FDA has approved a wide range of drug delivery systems for enhancing the efficacy and safety of chemotherapies. The most promising examples of these include Genexol-PM, a polymeric micellar formulation of paclitaxel, and Doxil, a liposomal formulation of Dox (129, 130). Various US-mediated materials and delivery platforms discussed here and summarized in **Table 1** are composed of ingredients that fall under the GRAS list. Therefore, the clinical development could be rapid for the drug delivery platforms that closely mimic the FDA-approved delivery systems. Secondly, clinical trials have been conducted on a few conjugates of FA and drugs (vinblastine and mitomycin C) using hydrophilic peptide spacers of varying lengths (131). The cancers included in these trials were small cell lung cancer, pituitary tumor, and OCs, however, the trial data have not yet been published. Notwithstanding the non-availability of the clinical trial data, concerning safety of the therapeutic formulation it is reassuring that FA has the regulatory approval for human application opening the door for US-mediated targeting of OCs. Lastly, targeting OCs with LHRH has been assessed in the clinical setting. AEZ-108, a conjugate of Dox with D-Lys^6^-LHRH showed a favorable safety profile in phase I clinical trial and in a proof-of-concept study on patients with platinum-refractory or platinum-resistant OC showed 52% clinical benefit rate calculated by adding complete response, stable disease and progressive disease data (132). Therefore, translation of LHRH and/or FA targeting strategies adopted to FDA-approved delivery platforms can undergo rapid clinical development for the treatment of OCs.

Besides the constituents of the formulations for US-mediated drug delivery, the safety of US per se is of utmost concern if prolonged and several cycles of high-intensity focused exposure is necessary for drug delivery. The potential of damage to normal tissue such as induction of any haemorrhage and its propagation in the US path, as well as time for complete reversibility from such damages need careful consideration before translating any US-mediated drug delivery technology to the clinic.

Finally, the clinical development of a new drug is heavily influenced by its econometry, which requires sophisticated sets of calculations due to the large number of variables involved. Most health economists believe that it would be extremely cost-effective to add a year to someone’s life expectancy for less than $3,000. In the case of cancer patients, one additional drug approval could increase 5-year survival by 2.4% (133). Based on this calculation, it is surmised that a rapid translation of, for instance, US-mediated LHRH and/or FA targeting techniques could be significantly more affordable in the treatment of OCs.

## Summary and future perspective

In this review, we focused on the most recent developments in US-mediated drug delivery strategies aimed at improving the treatment of OCs including refractory and drug-resistant types while reducing toxic effects. US-mediated targeting of OCs for therapeutic delivery made use of OC-specific markers (CA125 and CEA), receptors that are overexpressed in OCs (LHRH receptor and FAR), tumor hypoxia, and ovarian CSCs. Besides, blocking tumor angiogenesis by silencing VEGF, delivery of tumor suppressor protein (Sirt2), and enhancement of ICD by chemotherapeutic drugs by various US-mediated delivery strategies have shown promising results. The general delivery strategies consist of a combination of drug-loaded polymeric micelles and nano- or micro-emulsion droplets stabilized by biodegradable block copolymers consisting of polymer chains that are enzymatically or hydrolytically cleaved, thus generating soluble degradation products. Drug-loaded and echogenic polymeric micelles and nanoparticles have also been designed that upon injection extravasate through the defective tumor microvasculature and selectively accumulate in the tumor. Once inside the tumor, the accumulated bubbles coalesce and serve as long-lasting US contrast agents. Coupled with chemotherapeutic agents or biologics, these US contrast agents could serve the dual purpose of imaging and treatment of ovarian tumors. Future studies are required to determine and adjust the US responsiveness (cavitation qualities) of drug-loaded MBs based on the type of stabilizing copolymer used, bubble size, and therapeutic US parameters. Also, more aggressive OC lines that are derived from grade 3 tumors and have become drug resistant should be used for evaluating the efficacy of US-mediated treatments. Finally, the absence of head-to-head studies, which are ideal for comparing the efficacy between the treatments, prevents us from determining which is the most effective US-mediated drug delivery system.

There is considerable scope for developing new technologies using US with specific applications to treat OCs. One such approach would be to target the mechanotransduction pathways in ovarian tumors. Altered collagen cross-linking and the Rho-associated (ROCK) serine/threonine kinases have emerged as central regulators of the actomyosin cytoskeleton that underlie the mechanotransduction pathway in cancer cells that promote their migration to distant sites (134). The myosin-generated force has been shown to promote mesothelial cell displacement by OCs on their way to metastasis (135). US-mediated vehicle systems could be developed for delivery of β-aminopropionitrile, which prevents the formation of collagen cross-links, or the administration of Y27632 (a Rho/Rho kinase inhibitor), which prevents the formation of ECM cross-links or contractile actomyosin activity in OCs.

Another potential therapeutic use of US in OC is to deliver thermotherapy in combination with radio- or chemotherapy. Using MRI-guided targeted US, this method could selectively heat ovarian tumors to 40-43°C. To accomplish this, a thermotherapy delivery system might be developed based on a commercially available FDA-approved body-focused US transducer that establishes magnetic resonance thermometry parameters and identifies US beam synthesis and control approaches for conformal heating and temperature management. This technique administered in combination with conventional radio- and chemotherapy could significantly enhance the penetration efficiency, particularly for advanced OCs. Finally, a US-sensitive protein, transient receptor potential-4 which is a pore-forming subunit of a mechanotransduction channel and sensitizes neurons of *Caenorhabditis elegans* to US stimulus could be used for MB-independent targeting of chemotherapeutics. US-sensitive proteins constitute an emerging area of non-invasive manipulation of cells and tissues that is termed sonogenetics. Several recently identified mechanosensory channels including Piezo, degenerin family of ion channels, epithelial sodium channel (amiloride-sensitive), and two‐pore domain potassium (K2P) family of K^+^ channels can be tested whether further improvement in the logistics s US-mediated drug delivery is achieved.

## Author contributions

Y-CW: Data collection, writing a part of manuscript. J-YT: Data collection, writing a part of manuscript. Y-YH: Writing a part of manuscript, Table preparation. Y-FL: Writing a part of manuscript, figure preparation. S-YC: Data collection, writing a part of manuscript. F-JG: Conceptualization, supervision, reviewing the manuscript. All authors contributed to the article and approved the submitted version.

## Conflict of interest

The authors declare that the research was conducted in the absence of any commercial or financial relationships that could be construed as a potential conflict of interest.

## Publisher’s note

All claims expressed in this article are solely those of the authors and do not necessarily represent those of their affiliated organizations, or those of the publisher, the editors and the reviewers. Any product that may be evaluated in this article, or claim that may be made by its manufacturer, is not guaranteed or endorsed by the publisher.
